# Quantitative assessment of gait and neurochemical correlation in a classical murine model of Parkinson’s disease

**DOI:** 10.1186/1471-2202-13-142

**Published:** 2012-11-14

**Authors:** Xiao Hong Wang, Gang Lu, Xiang Hu, Kam Sze Tsang, Wing Hang Kwong, Feng Xia Wu, Hai Wei Meng, Shu Jiang, Shu Wei Liu, Ho Keung Ng, Wai Sang Poon

**Affiliations:** 1 Research Center for Sectional and Imaging Anatomy, Shandong University School of Medicine, 250012, Jinan, Shandong, China; 2 Division of Neurosurgery, Department of Surgery, Prince of Wales Hospital, The Chinese University of Hong Kong, Hong Kong, China; 3 School of Biomedical Sciences, The Chinese University of Hong Kong, Hong Kong, China; 4 Shenzhen Beike Cell Engineering Research Institute, Shenzhen, China; 5 Department of Anatomical and Cellular Pathology, The Chinese University of Hong Kong, Hong Kong, China

**Keywords:** Parkinson’s disease, Gait, MPTP, Tyrosine hydroxylase, Neurochemical correlation

## Abstract

**Background:**

Gait deficits are important clinical symptoms of Parkinson’s disease (PD). However, existing behavioral tests for the detection of motor impairments in rodents with systemic dopamine depletion only measure akinesia and dyskinesia, and data focusing on gait are scarce. We evaluated gait changes in the methyl-4-phenyl-1,2,3,6-tetrahydropyridine (MPTP)-induced C57BL/6 murine model of PD by using a computer-assisted CatWalk system. Correlations of gait parameters with tyrosine hydroxylase (TH) protein levels in the substantia nigra (SN) were also investigated.

**Results:**

The gait readouts, including the walking duration, variation of walking speed, step cycle, duty cycle, stance, initial dual stance, terminal dual stance, three- and four-point supports, and the base of support between hind limbs was noted to increase significantly one week after MPTP injection. In contrast, values of the stride length, cadence, swing speed, and diagonal dual support decreased substantially following MPTP treatment (p < 0.05). All of these changes lasted for three weeks after the last MPTP administration. Except for the stance in the fore limbs and the swing speed in the hind limbs, the gait variability in the PD mice showed a closer correlation with the protein levels of TH in the SN than the walking distances in the conventional open field test. Coordination parameters of the regularity index and step pattern were not affected in mice treated with MPTP.

**Conclusion:**

Data of the study suggest that the computer-assisted CatWalk system can provide reliable and objective criteria to stratify gait changes arising from MPTP-induced bilateral lesions in C57/BL6 mice. The extent of gait changes was noted to correlate with the expression of the biomarker for dopaminergic neurons. This novel analytical method may hold promise in the study of disease progression and new drug screening in a murine PD model.

## Background

Parkinson’s disease (PD) is a pervasive motor disorder resulting from the depletion of dopamine (DA) in the nigrostriatal region of the central nervous system [1]. Gait variability and postural instability are prominent as PD progresses [2]. Rhythmical walking of PD patients is hampered by short stride lengths and low velocities, which are related to reduction in the dopamine level [3,4]. The postural instability in PD patients often results in falls, when turning around or stepping up, leading to various injuries [5]. The motor deficits are the direct consequence of DA loss in the nigrostriatal system [1]. Tyrosine hydroxylase (TH) is the key enzyme in DA synthesis, as it converts tyrosine into levodopa, and is often used as a biomarker for dopaminergic neurons [6,7].

Methyl-4-phenyl-1,2,3,6-tetrahydropyridine (MPTP) is a neurotoxin being systemically administered to animals for experimental studies of PD [8,9]. It can induce neuropathological changes that mimic those of idiopathic PD in experimental animals, especially in C57/BL6 mice [10,11]. The behavioral tests used in acute, sub-acute, and chronic MPTP-induced animal models include the open field test [12,13], rotarod test [14,15], swimming test [16], and nest building test [17], which results may vary [18-20]. Experimental factors such as timing of testing (day or night), training intention, and testing environment, influence the readouts. Observable changes may be weak and the indicators of these tests, which are usually applied to unilaterally lesioned animals, may not be sufficiently sensitive to demonstrate the functional deficits in bilaterally injured animal models. Besides, animals are tested in an involuntary state and the test indicators, which are limited to either static or dynamic state, cannot adequately display both readouts simultaneously.

Gait analysis has already been described and applied to the assessment of movement disorders in PD [21-24]. Much effort has been made to analyze the gait in animal PD models, nevertheless there is a surprising lack of validation and characterization of gait variability in bilaterally lesioned animal models [23,25-32]. Alexander et al. investigated gait patterns and co-ordinations between limbs of rats subjected to unilateral 6-hydroxydopamine (6-OHDA) lesion by using footprint analysis and ladder walking test [26]. Chuang et al. evaluated gait patterns in a unilaterally 6-OHDA-induced rat model [33]. Fernagut et al. first introduced the manual measurement of stride length using foot prints in bilaterally MPTP-induced lesioned C57/BL6 mice [25]. Goldberg and co-workers assessed stride length, stride frequency, stride duration, variability in stride length, and paw area in MPTP-treated mice having been stressfully trained on treadmill apparatus [23]. Westin et al. investigated gait disorders in 6-OHDA-treated rats by a less commonly used bilateral induction method [34].

In this report we described a novel procedure to validate gait variability in a mouse model of sub-acute PD of bilateral lesion induced by MPTP. We evaluated the temporal expressions of TH protein in the SN and striatum of MPTP-treated mice and assessed the neuro-degeneration in the nigrostriatal regions by studying the correlation of TH expressions with changes of gait parameters.

## Methods

### Animals

Inbred strain of adult male mice C57BL/6 at aged 8–10 weeks and weighting 25–28 g were used in the study. They were kept in micro-isolator cages with access food and water *ad libium* in a temperature-controlled environment at 21°C and 12-hour cycle of light and dark. Animals were amenable daily to the researchers for one week prior to any experiment. Experimental procedures were performed according to the guidelines of the Animal Experiment Ethics Committee of the Chinese University of Hong Kong.

### Experiment design

**Figure 1:**
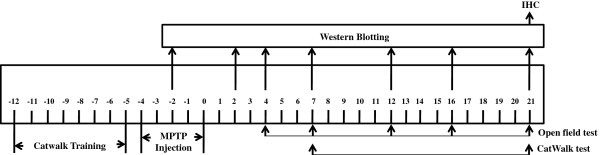
Flow chart of the study.

The design of the study is shown in Figure 1. C57BL/6 mice were allowed to familiarize with the CatWalk device daily and cross the runway in a consistent manner for one week before any experimentation. The mice were then randomly divided into two groups. MPTP-HCl (Sigma, St Louis, MO, USA) at 30 mg per kg body weight per day was injected intra-peritoneally into 50 mice for five consecutive days to induce PD. Normal saline in equal volume of MPTP-HCl was injected into 45 control mice. Upon completion of the administration of either MPTP or normal saline, mice were subjected to computer-assisted CatWalk tests on day 7 and day 21 and open field tests on days 4, 7, 12, 16, and 21.

Western blotting was performed to analyze TH expressions in the SN and striatum of mice two days before and on days 2, 4, 7, 12, 16, and 21 after the course of MPTP administration. Immuno-histochemical staining of TH was conducted to assess the loss of dopaminergic neurons in the SN and striatum of the mice three weeks post-injection of MPTP.

### Gait analysis

Gait of unforced moving mice was analyzed with the CatWalk XT (Noldus Information Technology, Wageningen, Netherlands). Details have been described previously [35]. CatWalk XT consists of a hardware system of a long, glass walkway plate, illuminated with green light, a high-speed video camera, and a software package for quantitative assessment of animal footprints. Green light is reflected within the glass except at points being touched. It scatters and illuminates the contact area. The intensity of the area of illumination, which is proportional to the exerted pressure, is digitally captured by the video camera and analyzed.

Mice were allowed to walk across the glass walkway in an unforced manner at least six times a day on day 7 prior to injection with either MPTP or normal saline. Mouse tracks that were straight without any interruption or hesitation were treated as successful runs. Runs with any wall climbing, grooming, and staying on the walkway were not analyzed. Mice that failed the CatWalk training were excluded from the study. An average number of 8 replicate crossings (range: 4–6) made by each mouse was recorded. The CatWalk software was used to analyze crossings that had at least five cycles of complete steps. Table 1 shows the definition of the gait and co-ordination parameters used in this study.

**Table 1:** Definitions of gait parameters

**Parameter**	**Definition**
Run duration	Time of finishing an entire run in second
Maximum area	The print area of paw when the braking phase turns into propulsion phase
Maximum intensity	Maximum pressure of paw contacting floor
Stride length	The distance between two consecutive travels in the same paw
Swing duration	The duration in seconds of no contact of a paw with the glass plate
Swing speed	The ratio of stride length to swing duration
Stance	The time duration in seconds of paws in contact with glass plate
Step cycle	The time in seconds between two consecutive initial contacts of the same paw
Duty cycle	The percentage of stance over the sum of stance and swing duration
Cadence	Steps per second in a trial
Base of support	Distance between fore limbs or hind limbs at maximum area
Initial dual support	The first time in a step cycle of a hind paw that the contralateral paw also makes contact with the glass plate
Terminal dual support	The second step in a step cycle of a paw that the contralateral paw also makes contact with the glass plate
Diagonal dual support	
Three-point support	
Four-point Support	The relative duration of simultaneous contacts of limbs with the glass plate
Step Pattern	Configuration of right (R), left (L), front (F) and hind (H) limbs
Alternate Aa: RF-RH-LF-LH; Ab: LF-RH-RF-LH	
Cruciate Ca: RF-LF-RH-LH; Cb: LF-RF-LH-RH	
Rotary Ra: RF-LF-LH-RH; Rb: LF-RF-RH-LH	
Regularity Index	The number of normal step sequence patterns relative to the total number of paw placements

### Open field test

Spontaneous locomotor activity in mice was measured using the open field test. Mice were allowed to adapt to the environment for two hours prior to testing. They were then placed individually facing the same wall of a white square box measuring 50 × 50 × 25 cm and kept for 10 minutes under normal lighting. Movements and trajectories of mice were video-taped and analyzed by the Videomex-One software (Columbus Instruments, USA). The box was cleaned with water and 70% alcohol after each testing to remove the body scent, which could be a cue to move for mice in subsequent evaluations. Eight mice per study arm and time point were recruited.

### Tissue processing

Before and upon completion of MPTP injection on day −2 and days 2, 4, 7, 12, 16, and 21, respectively, four mice were anesthetized, their thoraxes were cut open, and they were trans-cardiacally perfused with physiologic saline and 4% paraformaldehyde in 0.1 M of phosphate-buffered saline. Heads were decapitated. Brains were rapidly removed and fixed in 4% paraformaldehyde for two days at 4°C, and allowed to further fix in 15%, 20%, and 30% sucrose solution.

The dissecting protocol of Jackson-Lewis and Przedborski was adopted to maximize the isolation of ventral midbrain containing the substantia nigra [36]. The brain was quickly removed and put into a chilled mouse coronal brain matrix (World Precision Instruments Inc., USA). A 2-mm-thick posterior-anterior segment was dissected from the posterior aspect of the cerebral peduncles. The caudal aspect of the dissected section was immediately placed on the disk with the ventral aspect facing the researcher. After the middle brain was separated, the substantia nigra-rich tissue was isolated by cutting at a third of the way from the dorsal midbrain with a blade slanted at a 45° angle toward the researcher. The striatum was dissected from the brain immediately. All of the brain tissues were stored at −80°C until analysis.

### Western blot analysis

Brain tissues was homogenized in ice-cold lysis buffer containing 50 mM Tris–HCl at pH 6.8, 50 mM NaCl, 1% Triton X-100, 1 mM PMSF, 5 μg/ml of aprotinin, 1 μg/ml of leupeptin and 1 μg/ml of prepstetin (ICN Bio Medical Products, Costa Mesa, CA). The lysates were centrifuged at 12,000 x *g* for 30 minutes at 4°C to separate debris. The protein concentration was assayed using a BIO-RAD DC protein assay kit (BioRad, Hercules, CA). Ten μg of protein was used for the western blot analysis.

Protein samples were electrophoresed on 10% SDS-PAGE and blotted onto nitrocellulose membranes (BioRad, Hercules, CA). Having blocked for an hour at room temperature with 20 mM Tris–HCl at pH 7.5, 137 mM NaCl, 0.05% Tween 20 and 5% non-fat milk (BioRad, Hercules, CA). proteins on membranes were incubated with polyclonal rabbit anti-TH (1:3,000, clone AB152, Chemicon International, USA), polyclonal goat anti-β-actin (1:1,000, Santa Cruz, USA) overnight at 4°C. Membranes were washed three times and then stained with fluorescein isothiocyanate-conjugated secondary antibody (1:20,000, Rockland, USA) for an hour at room temperature. Upon completion of washings, green fluorescent signals were visualized using the Odyssey® imaging system (LICOR Biosciences, version 3.0). The optical density was read using the Odyssey system.

### Immunohistochemical staining of TH

Cryostat sections of brain slices in cryo-mountant were cut 25 μm thick. Floating sections on 0.01 M phosphate buffered saline were exposed to 0.25% Triton X-100 and 3% H_2_O_2_, and then blocked in 2% bovine serum albumin. They were then incubated sequentially with rabbit polyclonal anti-TH antibody (1: 1,000, clone AB152, Chemicon International, USA), biotinylated goat anti-rabbit antibody (1:200, Vector Laboratories, USA) and horseradish-peroxidase-conjugated avidin-biotin complex (Vector Laboratories, USA). Sections were developed with a DAB kit (Zymed Laboratories Inc, USA). Dark brown signals derived from sections mounted on glass slides were captured and analyzed using a microscope Axioplan 2 (Zeiss, Germany) equipped with a documentation system.

Unbiased stereological counting method was used to enumerate the DA neurons [37]. Every sixth section of the SN was analyzed by means of the optical fractionator and a computer-assisted stereological Olympus Toolbox system version 2.1.4. The SN of each hemisphere was first delineated using a 4x objective. A square grid of 150 × 150 μm was randomly super-imposed with and a 100 × 100 μm square dissector counting chamber placed on the first counting area of the image and then systemically moved through all of the counting areas until the entire delineated section was counted. The striatum of each section was located under the same identical condition. Analyses of the TH-immunoreactive profiles were restricted to the SNpc and thus excluded the ventral tegmental area. The optical density of the TH-immuno-reactive fibers was evaluated by the software Metamorph 6.3 on 9–11 sections spanning the striatum. The background signal captured from the corpus callosum was subtracted from the readout of the optical density of each striatal section. Scorings of TH-immuno-reactivity and optical density were performed by two researchers in a blind manner. Results derived from 9–11 sections were averaged.

### Statistical analysis

All the data were expressed as mean ± standard error of mean (SEM). One-way ANOVA was used for the statistical evaluation of gait changes in the CatWalk test. Repeated-measure one-way ANOVA was used to statistically analyze the path lengths in the open field test at various time intervals after MPTP injection. The correlations of the TH protein levels in the SN with the motor parameters in the CatWalk test and open field test were evaluated by the Pearson’s product–moment correlation coefficient. Differences in the expression levels of TH protein at different time points were analyzed by ANOVA and the Student–Newman–Keuls post hoc test. Quantitative differences of TH-immuno-reactive cells in SN were evaluated using the Student’s t-test. A *p* value ≤ 0.05 was considered as statistically significant. All of the data were analyzed using the SPSS 17.0 software (SPSS Inc., Chicago, USA).

## Results

### Catwalk test

**Figure 2:**
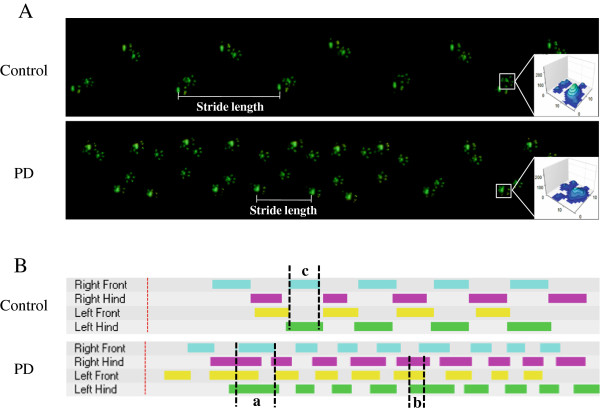
Representative illuminations of footprints.

**Figure 3:**
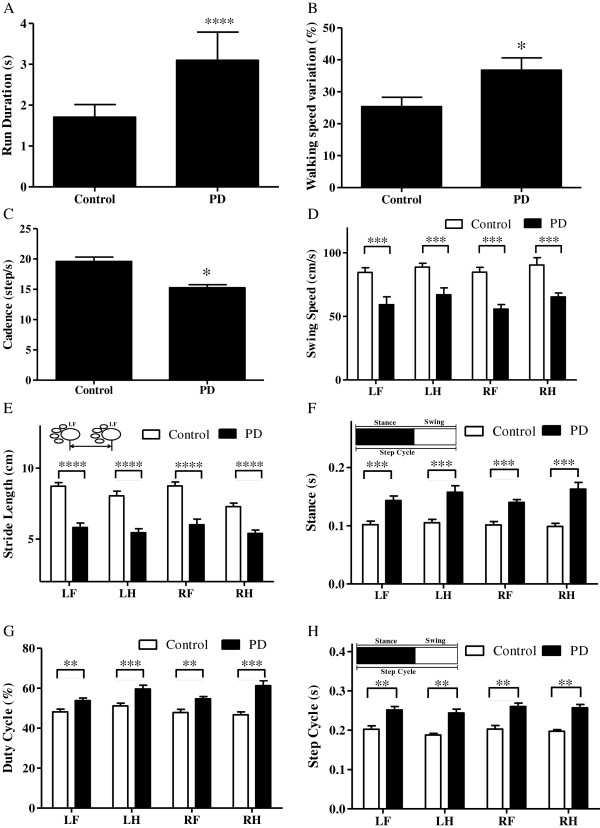
Gait variability three weeks post-MPTP administration.

Gait of mice was evaluated with CatWalk system at one week and three weeks upon completion of the administration of either MPTP or normal saline. A decrease in the stride length and an increase in variation of stance and swing were noted in the MPTP-treated mouse compared to the control mouse. Besides, A fall in diagonal dual support and an upsurge of four- and three-point supports were evident (Figure 2). Abnormal gait persisted over the three-week study period. A mean ± SEM of walking duration taken by eight mice on day 21 post-MPTP treatment was significantly longer than that of eight control mice having normal saline injection (time in seconds: MPTP-induced PD mice vs. control mice; 3.099 ± 0.689 seconds vs. 1.708 ± 0.308 seconds; *p ≤* 0.01, Figure 3A). The extent of variation in walking speed of MPTP-treated mice was more profound than that of the control mice (MPTP-induced PD mice vs. control mice; 34.92 ± 3.61% vs. 24.44 ± 2.49%, *p* = 0.019, Figure 3B). The cadence of MPTP-treated mice dropped significantly (steps per second: MPTP-induced PD mice vs. control mice; 15.18 ± 0.52 vs. 19.19 ± 0.68; *p* ≤ 0.05, Figure 3C).

**Figure 4:**
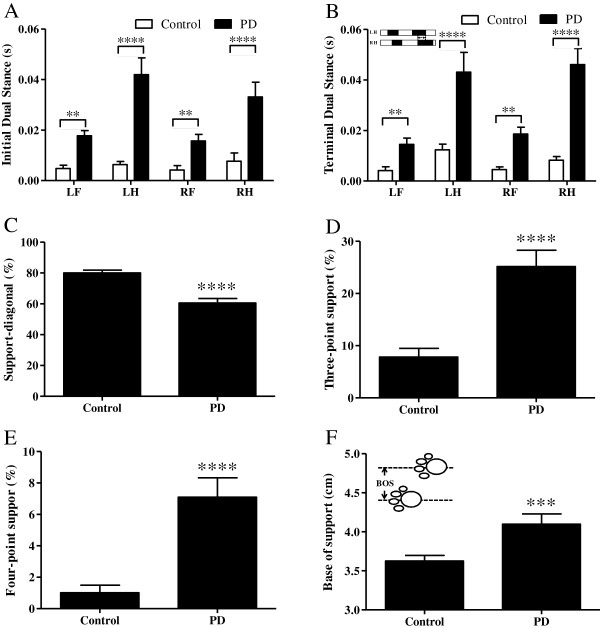
Gait changes three weeks post-MPTP administration.

The swing speeds of paws deteriorated markedly (Figure 3D) and the stride lengths of limbs decreased significantly (Figure 3E), whereas the stances, duty cycles, and step cycles of paws increased substantially (Figures 3 F–H). The initial dual stance and terminal dual stance of paw were also prolonged in MPTP-treated mice compared with those of control counterparts (Figure 4A–B). A relative decrease in diagonal dual support was noted in MPTP-treated mice (incidence of diagonal dual support: MPTP-induced PD mice vs. control mice; 59.76 ± 3.06% vs. 80.52 ± 1.77%, *p* ≤ 0.0001, Figure 4C). Significant increases of three-point support and four-point support were noted in MPTP-treated mice (incidence of three-point support: PD mice vs. control mice; 25.99 ± 3.26% vs. 8.07 ± 1.62%; *p* ≤ 0.0001; Figure 4D, incidence of four-point support: PD mice vs. control mice; 7.33 ± 1.3% vs. 1.24 ± 0.47%; *p* ≤ 0.0001; Figure 4E). The bases of support between hind limbs were relatively longer (PD mice vs. control mice; 4.12 ± 0.08 cm vs. 3.63 ± 0.06 cm; *p* ≤ 0.0001; Figure 4F).

On the other hand, readouts of gait parameters of maximum pressure intensity, maximum contact area, and swing duration of paws of MPTP-treated mice and control mice are comparable (data are not shown). There was also no significant difference in inter-limb coordination among PD mice and normal mice.

Step patterns and regularity index are indices of coordination, and in these two measures there were no differences between the treated group and the controls. It may be that the motor dysfunction induced by the nigrostriatal lesion in this study did not affect the coordination of the tetrapods. This supposition is supported by the results of Chuang and his colleague [33].

### Open field test

**Figure 5:**
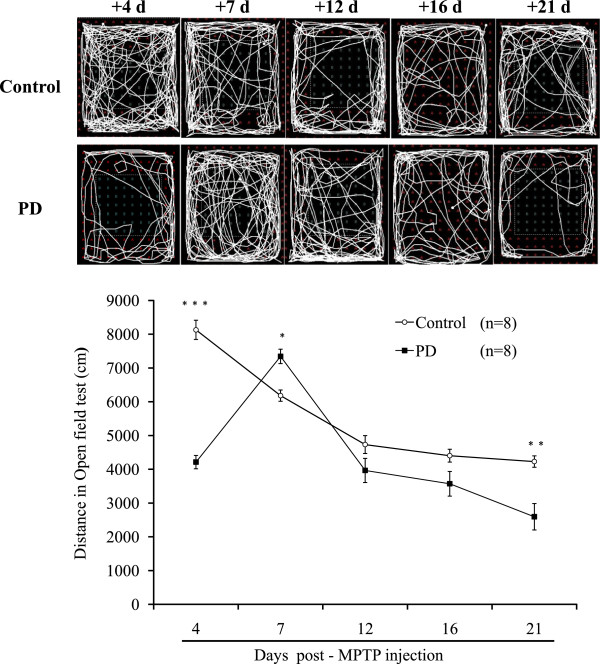
Representative trajectories and locomotor activity in open field test.

The locomotor activity was measured by means of the open field test to investigate the time course of changes in spontaneous activity induced by the MPTP. Repeated-measure one way ANOVA revealed the effect of the number of times of repetitive tests (F4,11 = 106.6; p < 0.001) and the treatment interaction (F4,11 = 42.865; P < 0.001), indicating that MPTP treatment affected spontaneous activity compared with saline treated mice (Figure 5). The distances travelled by mice on day 4 and at three weeks after the last injection of MPTP were significantly less than those of the vehicle-treated mice (*p* < 0.001).

### Expression and correlation of TH with gait variability

**Figure 6:**
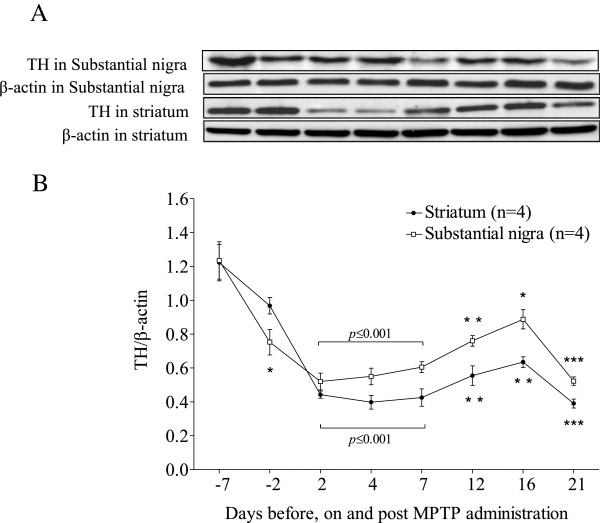
Western immunoblotting of tyrosine hydroxylase (TH) in the substantia nigra and striatum of mice before, on and post MPTP administration.

A significant fall in TH was evident in both substantia nigra and striatum of mice starting on day 2 of MPTP administration, followed by a slight rebound at two weeks. TH then remained at the lowest levels on three weeks post-MPTP injection. It was noted that the down-regulation of TH by MPTP in the striatum was more profound than that in the substantia nigra (Figure 6).

Table 2 shows the significantly positive correlations of the levels of TH in the substantia nigra of MPTP-treated mice with readouts derived from CatWalk tests of diagonal dual support, stride length in all limbs, and swing speed in the forelimbs three weeks post MPTP administration. Substantially negative correlations of TH levels with stance in the hind limbs, step cycle, duty cycle, initial dual stance, terminal dual stance, three-point support, four-point support, walking speed variation, cadence,and base of support between hind limbs were noted. On the other hand, the levels of TH correlated poorly with the trajectories in the open field tests.

**Table 2:** Correlation of motor deficits with tyrosine hydroxylase expression in substantia nigra three weeks post MPTP administration

**Parameters**	**Pearson correlation coefficient**	***P*-value**	**Parameters**	**Pearson correlation coefficient**	***P*-value**
Stance			Duty cycle		
LF	-0.183	0.235	LF	-0.444	0.003
LH	-0.420	0.003	LH	-0.594	0.0001
RF	-0.191	0.215	RF	-0.422	0.004
RH	-0.447	0.002	RH	-0.717	0.0001
Stride length			Initial dual support		
LF	0.759	0.0001	LF	-0.385	0.01
LH	0.725	0.0001	LH	-0.466	0.001
RF	0.660	0.0001	RF	-0.354	0.019
RH	0.568	0.0001	RH	-0.419	0.001
Swing speed			Terminal dual support		
LF	0.393	0.008	LF	-0.455	0.002
LH	0.252	0.099	LH	-0.385	0.010
RF	0.359	0.007	RF	-0.497	0.001
RH	0.288	0.058	RH	-0.357	0.022
Step cycle			Diagonal dual support	0.677	0.0001
LF	-0.463	0.001	Three-point support	-0.589	0.0001
LH	-0.301	0.046	Four-point support	-0.455	0.002
RF	-0.453	0.002	Speed variation	-0.469	0.001
RH	-0.368	0.014	Cadence	-0.346	0.021
Distance in open field test	0.206	0.358	Base of support between hind limbs	-0.569	0.001

LF: left front, LH: left hind, RF: right front, RH: right hind.

### Histological markers of nigrostriatal degeneration induced by MPTP

**Figure 7:**
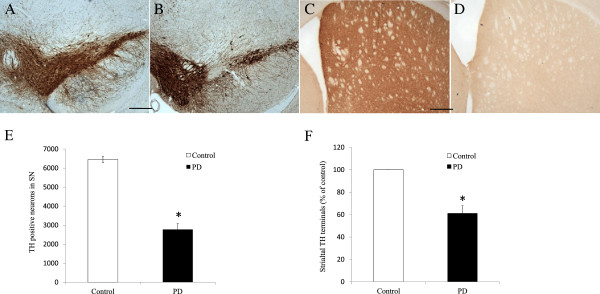
Enumeration of neurons expressing tyrosine hydroxylase (TH) in the substantia nigra and striatum of mice three weeks post-MPTP administration.

Immuno-histochemical staining of TH demonstrated a substantial loss of immuno-reactivity in the SN and striatum of mice three weeks post-MPTP administration (Figure 7). Unbiased stereological counting displayed a significant loss of up to 57.04% compared with the control group on the 21^st^ day after the last injection of MPTP (*p <* 0.05). Injection with MPTP also resulted in a great depletion of TH-ir in the striatum of up to 61.32% of that in control group on the 21^st^ day post-treatment. Data suggest that deprivation and denervation of TH-immuno-reactive neurons and terminals might be attributed to the gait variability experienced by MPTP-treated mice.

## Discussion

### Quantitative assessment of gait

In this study, we developed a time-efficient and animal-friendly CatWalk test of motor functions in an MPTP-induced PD mouse model. Both static and dynamic parameters of gait variability were collected simultaneously. Static parameters of stance, print length, print width, print area, maximum contact intensity, and maximum contact area are based upon contacts of individual paw with the glass plate of walkway. Dynamic parameters are walk duration, variation of walk speed, swing speed, and base of support between limbs. The study showed that the CatWalk test was able to sensitively assess gait disorder and inter-limb coordination deficit in a mouse model of PD with bilateral lesion of DA neurons.

There are many behavioral tests in MPTP-induced PD mouse model. These have to be robust and sensitive, in order to detect functional changes, days and even weeks after challenge by a neuro-toxin. The widely employed rotarod test is a feasible means to analyze coordination disorders in a bilaterally lesioned PD mouse model, but it is insensitive. It was noted that the progress of pre-analytical training of animals may recruit DA in the striatum [38]. The pole-, grid- and nest-building tests are sensitive to striatal DA level in MPTP-treated mice, but are heavily dependent on the skilled manipulation of fore-limbs of the animal being studied [17,39,40].

The open field test is the most commonly used method to evaluate motor deficits because of its ease and rapidity of use and rapidity in animals following MPTP insult [12,41-45]. We conducted open field test to study motor deficits in mice three weeks after induction of sub-acute neural injury and applied the open field test as the control in the evaluation of the CatWalk test. Readouts of the open field test in the study agree with previous reports of significant motor deficits in mice a few days after MPTP injection [43,44]. A significant upsurge of trajectories was also noted in mice one week post-MPTP administration compared to those read on day 4 and that of control mice on day 7. The scenario might be attributable to an increase of DA metabolism and fluctuation of catecholamines such as 5-hydroxytryptamine in the midbrains of mice induced by dopaminergic neurotic and MPTP and a booster of muscle strength to balance posture instability [46-49].

Although open field test is a friendly test, the time points to detect locomotor changes in animals are limited. Here, we could only apply the time points of the 4 day and three weeks after MPTP injury for the functional evaluation in the open field test. The stability of behavioral performance of animals in the open field is vulnerable to the toxic effects of MPTP on biogenic amines inside and outside the brain [44], administration regimens and dosages of MPTP [48,50] and environments.

The computer-assisted CatWalk test paralleled the motor function assessment in the open field test by collecting static and dynamic parameters in non-stressed rodents to investigate gait variability and coordination changes simultaneously. The stride length, run duration, stance, step cycle, duty cycle, swing speed, and cadence can be used to assess gait changes. The base of support between limbs, initial dual stance, terminal dual stance and diagonal dual support, three-point support and four-point support reflects the posture alterations. The regularity index and step sequence evaluates inter-limb coordination. Animals therefore do not need to undergo any complex process of training. They are allowed to simply run freely along the glass walkway four to six times. Data from the study show that their abnormal gait , in terms of stride length, duty cycle, dual diagonal support, three-point support, four-point support, and base of support between limbs persisted up to three weeks post-MPTP administration. The parameters of inter-limb coordination, regularity index, and step pattern showed no significant difference in MPTP-treated mice, compared to those of control mice. The extent of MPTP-mediated nigrostriatal lesion might not elicit any observable dis-coordination of tetrapod [33]. The observation may also be related to stronger postural adjustment in quadrupeds than in bipeds.

### TH profiling at time of MPTP insult

MPTP-mediated dopaminergic cell loss occurred predominantly in the SN, whereas DA neurons in the ventral tegmental area were less affected [8,51].

The remarkable reproducible neuroplasticity of the nigrostriatal dopaminergic neurons after MPTP injury has been reported in mice [52]. The decrease in the expression of TH protein was noted to correlate with the loss of DA neurons in the SN due to acute MPTP injury [9]. It is likely that the decline in striatal dopamine content was due to the decrease in the expression of TH protein as a consequence of MPTP lesion. The rescue of TH protein is one of the determinants for regaining striatal dopamine in surviving nigrostriatal dopaminergic neurons [40-42]. Readouts of western blotting in the study demonstrated that protein levels of TH in the SN and striatum appeared to rebound two weeks post-MPTP administration; however the expressions were still significantly lower than those derived from intact SN and striatum.

The study data were in line with those reported previously by Ookubo et al. [53]. The TH protein level fell to the lowest extent after three weeks. The stable lesion in PD mice can serve as a proxy for Parkinsonism in mice, but not for the assessment of MPTP neurotoxicity. The up-regulated expression of TH protein expression two weeks after MPTP injury might have a robust compensatory increase resulted by surviving nigrostriatal dopaminergic neurons against the neurotoxicity of the MPTP [54,55]. The study data suggest that the open field test is insensitive to the extent of DA lesion except on day 4 and three weeks post-MPTP challenge.

### Translational investigation of gait variability in MPTP-treated mice

The study provided a thorough analysis of the very fine locomotor aspects in a mouse model of sub-acute PD mediated by MPTP. The CatWalk test revealed detailed gait changes in mice at one week and three weeks post-MPTP challenge. That stride length decreased markedly in MPTP-treated mice may be the result of an increase in muscle rigidity and hypokinesia [40,56,57]. Results of the study were in accordance with previous studies [23,27,34,58] and reports on PD patients [59-61], suggesting that stride length serves as a successful translational parameter for PD.

Bradykinesia is one of the major hallmarks of PD [59,62]. Run duration, speed variation, swing speed, and step cycle are parameters related to velocity in mobility analysis. Using the CatWalk analysis system, we identified a significant increase in walking duration accompanied with a great variation in the walking speed in MPTP-treated mice. The greater variation in walking speed, which is similar to the fractal-like gait in PD, suggests possible marked inconsistencies in the timing of gait and inadequate postural adjustments [63-65]. The step cycle was increased in the MPTP-treated mice of the study due mainly to the longer contact between the paws and the glass plate. Results of the study were in line with a previous gait assessment in a bilateral 6-OHDA rat model [34]. Gait cadence decreased significantly in MPTP-treated mice. However, an increase in step frequency was noted in PD [66,67]. Cadence varies directly with stride length and inversely with the step cycle. The decrease in stride length noted in MPTP-treated mice of the study might not counter-balance the increase in the step cycle. As a consequence, the cadence decreased in MPTP-treated mice.

Support, either temporal or spatial, is another aspect related to gait in PD. The significant increases of initial dual stance, terminal dual stance, three-point support, and four-point support and the substantial decrease in diagonal dual support in the study suggested an increased duration of the postural phase in the MPTP-treated mice. The study data agree with reports on a longer double limb support time in PD, which may stem from the long time taken to prepare for the generation of propulsive forces [3,68]. Results are in line with delays of freezing of gait or gait hesitation in PD [69-71]. The increased duration of the postural phase and the decrease in propulsive forces during the postural and movement phases resulted in a shorter step length and a slower step velocity. The spatial parameters of base of support between hind limbs increased substantially in MPTP-treated mice but there was no obvious difference in base of support between fore limbs. This might be related to hind limb rather than fore limb compensation, at least in part, playing a role in the gait instability of MPTP-treated mice in the study. On the other hand, the study data contrast with the findings of Westin et al. in which the base of support between fore limbs decreased significantly after 6-OHDA injury. This might be attributed to the difference in injured cerebral areas of animals in the two studies [34].

Interlimb coordination parameters of regularity index and step pattern were also studied. There are three categories in the step patterns: cruciate (Ca, Cb), alternate (Aa, Ab), and rotary (Ra, Rb). The ‘Ab’ alternate pattern is the most common step cycle, constituting 80% to 95% of the total step cycles in intact rodents, the other patterns are very rare in intact animals. The Ab pattern remains dominant in the Parkinson’s disease animal model [72]. In this study, there was a slight but not significant decrease in the incidence of this step pattern in mice after MPTP injury. This result is supported by Chuang [33], and may be related to stronger postural adjustment in quadrupeds than in bipeds.

### Correlation between the gait parameters and TH levels in the SN

Various strategies have been employed to verify the correlation between the motor deficits and the impairment of the nigrostriatal system. Here, we used correlation analysis of the motor parameters and TH protein levels in the SN. Compared with the travel distance measured in the open field test, parameters of the run duration, stride length, duty cycle, swing speed, base of support between hind limbs, three-point support, and four-point support in the CatWalk test were much more strongly correlated with the SN TH protein level. This is one of the first studies to apply correlation analysis to gait measures and TH protein levels in the SN to further confirm gait and posture deficits in the classic sub-acute MPTP PD model.

## Conclusion

This study reports novel and low-stress method of analyzing the motor functions of mice, which may be applied to monitor gait variability related to disease progression and treatment strategy.

## Competing interests

The authors declare that they have no conflicts of interests.

## Authors’ contributions

X Hu, G Lu, and XH Wang carried out the CatWalk and open field tests, collected the data, and drafted the manuscript. WS Poon, SW Liu, and KH Kwong contributed to the study design, discussed the results, and revised the manuscript. FX Wu, HW Meng, and KS Tsang performed the Western Blotting and histological analyses. FX Wu, S Jiang, and HK Ng carried out the model establishment and evaluation. All authors read and approved the final manuscript.
